# Superiorly Plasticized PVC/PBSA Blends through Crotonic and Acrylic Acid Functionalization of PVC

**DOI:** 10.3390/polym9030084

**Published:** 2017-03-01

**Authors:** Arturo Salazar Avalos, Minna Hakkarainen, Karin Odelius

**Affiliations:** Department of Fibre and Polymer Technology, KTH Royal Institute of Technology, SE-100 44 Stockholm, Sweden; aasa2@kth.se (A.S.A.); minna@kth.se (M.H.)

**Keywords:** PVC, poly(butylene succinate-*co*-adipate), polyester, functionalization, blend, plasticizer, acrylic acid, crotonic acid

## Abstract

Superior plasticization efficiency was achieved by a grafting from functionalization of the PVC backbone. This was deduced to a synergistic effect of internal plasticization and improved intermolecular interactions between PVC and an oligomeric poly(butylene succinate-*co*-adipate) (PBSA) plasticizer. A mild grafting process for functionalization of the PVC chain by crotonic acid (CA) or acrylic acid (AA) was used. The formation of PVC-*g*-CA and PVC-*g*-AA was confirmed by FTIR and ^1^H NMR. Grafting with the seemingly similar monomers, CA and AA, resulted in different macromolecular structures. AA is easily homopolymerized and long hydrophilic poly(acrylic acid) grafts are formed resulting in branched materials. Crotonic acid does not easily homopolymerize; instead, single crotonic acid units are located along the PVC chain, leading to basically linear PVC chains with pendant crotonic acid groups. The elongation of PVC-*g*-CA and PVC-*g*-AA in comparison to pure PVC were greatly increased from 6% to 128% and 167%, respectively, by the grafting reactions. Blending 20% (*w*/*w*) PBSA with PVC, PVC-AA or PVC-CA further increased the elongation at break to 150%, 240% and 320%, respectively, clearly showing a significant synergistic effect in the blends with functionalized PVC. This is a clearly promising milestone towards environmentally friendly flexible PVC materials.

## 1. Introduction

Applying green chemistry to polymers entails e.g., replacing petroleum with renewable resources, developing methods to create materials under benign conditions, and ensuring that the materials are non-toxic during usage as well as at end-of-life. This effort will be essential for reaching a sustainable society. However, replacing all current petroleum-based materials with green alternatives is not feasible in the short run. We need to have a long-term strategy and to proceed step by step. Poly(vinyl chloride) (PVC) is one of the most common and versatile polymeric materials with applications varying from rigid pipes and window frames to floorings, wallpapers, cables, packaging and medical products. This wide range of products is possible due to the relatively easy material modification by blending with additives or other polymers. The need for plasticizers to produce flexible PVC products has, due to the large production volumes, become one of the largest environmental and health issues connected to plastic materials. Phthalate esters are still the most common PVC plasticizers, even though the use of some of the traditional phthalate esters is now restricted in applications such as medical products and toys. These phthalate esters can migrate from the materials during use [1]. They have also been found in most environments, in domestic foods and wastes, in animals and humans, and they exhibited potential toxic and biological effects when transferred to humans or animals [2,3]. Finding green alternatives and preventing or minimizing plasticizer migration is, thus, important both to avoid a release of plasticizers into the environment as well as to prohibit premature loss of function.

The demand for alternative plasticizers has generated much research activity concentrating on identifying safe, environmentally friendly, and effective plasticizers [4]. Polycaprolactone [5], poly(butylene adipate) [6,7], succinate esters [8], isosorbide esters [9], glucose esters [10], esterified fatty acids [11], citric acid [12], cardanol derivatives [13] and ionic liquids [14] are just a few among the many evaluated compounds. The effect of macromolecular architecture on plasticization efficiency and migration resistance has been investigated in several studies [15,16,17], as well as plasticizers anchored on the surface of nanoparticles [18]. In addition, processes have been developed to prevent plasticizer migration using surface modification by coatings, crosslinking or grafting, or by utilizing migration inhibitors, such as encapsulated β-cyclodextrin [19,20] in traditional PVC/phthalate blends.

An appealing approach for migration-resistant, flexible PVC products has been presented through covalently bound plasticizers resembling the traditional phthalate plasticizers in chemical structure [21]. In one approach, covalent attachment of thiol-functionalized phthalates provided a permanent plasticizing effect with no migration into heptane. However, the plasticization efficiency of the bound plasticizers was lower than that of the traditional free-phthalate plasticizers. Using similar thiol-coupling chemistry, the group has more recently shown that covalently attached hydrophilic PEO-PPO have similar plasticization efficiencies as PVC/di-2-ethylhexylphthalate (DOP) mixtures as determined by their similar change in *T*_g_ [22]. Successful strategies to synthesize PVC bearing primary amine groups [23] and functional nucleophiles [24] have also recently been shown and have the possibility to broaden the application area of PVC even further. In another approach, PVC was azide-functionalized, after which a copper-free click reaction was performed, creating a phthalate-mimicking plasticizer covalently attached to the PVC backbone [25]. A biobased, cardanol-derived plasticizer was covalently attached to the backbone of PVC also by a click reaction, and the material showed a decrease in glass transition temperature and nearly no migration [26].

In addition to grafting PVC with molecules resembling traditional plasticizers, grafting chains or single groups that can break the secondary interactions between PVC chains and function as internal plasticizers at the same time as they increase the intermolecular interactions between the PVC and the plasticizer could be an attractive approach, especially if these groups can be attached by robust and environmentally friendly methods suitable for upscaling to industrial applications. Previously elegant controlled radical polymerization methods to graft methacrylates and styrene from chloroacetate units or from structural defects on the PVC main chain have been presented [27,28]. However, these methods are not translatable to e.g., acrylic acid, which can be derived from renewable resources, and their upscalability could be challenging. Several papers have presented PVC surface modification by free radical grafting of acrylic acid to obtain hydrophilic surfaces suitable for membranes or biomedical applications [29,30]. One approach utilized a two-step process: first, the physisorption of azobisisobutyronitrile (AIBN) onto the surface of PVC; second, the radical graft polymerization of the hydrophilic monomers onto the polymer surfaces. The thickness, roughness and chemical composition of the grafted layer was determined, and the method could be applied even to complex structures [29].

Instead of just grafting on the surface, a simple free-radical bulk modification of the PVC chains with compounds containing carboxylic acid groups could break the strong secondary bonding between the PVC chains and result in internal plasticization to yield self-plasticized PVC. Superior plasticization effect could be obtained by combining this internal plasticization with addition of miscible oligomeric plasticizers that, due to, for example, their size and architecture have a reduced migration rate [7,15,17]. Thus, we applied a straightforward method to covalently graft crotonic acid (CA) units and acrylic acid (AA) grafts from the main chain of PVC to achieve a self-plasticized effect. We hypothesized that monomeric functionalization (CA) and longer grafts yielding a branched PVC structure (AA) would be achieved due to the inherent differences in the reactivity’s of the two monomers and that the effects on PVC properties would be determined by graft structure and amount. A grafting-from functionalization methodology, where polymeric chains or monomer units are grafted from the main chain of PVC, provides further possibilities to increase intermolecular interactions between the functionalized PVC chains and added plasticizers, leading to further improvement in plasticization efficiency and migration resistance.

## 2. Materials and Methods

### 2.1. Materials

Poly(vinyl chloride) (PVC) (Sigma-Aldrich; *M*_n_ = 55,000 and *M*_w_ = 97,000) was used as received. 1,4-Butanediol (Bu) (99%, Riedel-de Haën, Göteborg, Sweden), adipic acid (AAc) (99%, Fluka, Göteborg, Sweden), succinic acid (SAc) (99%, Fluka, Göteborg, Sweden), titanium isopropoxide (TIP) (99.99%, SigmaAldrich, Stockholm, Sweden), benzoyl peroxide (BPO) (70%–75%, Acros Organics, Waltham, MA, USA), and methanol (99.93%, SigmaAldrich, Stockholm, Sweden) were used as received for the synthesis of oligomeric polyester plasticizer without further purification. Acrylic acid (AA) (90%, Alfa Aesar, Ward Hill, MA, USA) was purified by vacuum distillation at 40 °C prior to use. Crotonic acid (CA) (98%, SigmaAldrich, Stockholm, Sweden), cyclohexanone (99%, SigmaAldrich, Stockholm, Sweden) and dichloromethane (DCM) (99.8%, SigmaAldrich, Stockholm, Sweden) were used as received for the PVC modification. Tetrahydrofuran (THF) (99.8%, HPLC grade, Emsure, Darmstadt, Germany) was used for the film preparation. Dimethyl sulfoxide-D_6_ (DMSO) (99.9%, Larodan Fine Chemicals AB, Stockholm, Sweden), chloroform-d (CDCl_3_) (99.8%, Cambridge Isotope Laboratories, Tewksbury, MA, USA) with silver foil were used for the NMR characterization.

### 2.2. Synthesis of the Polyester Plasticizer

The synthesis of PBSA was carried out in a two neck round-bottom reaction vessel equipped with a magnetic stirring bar. Bu and a mixture of the acids succinic acid:adipic acid = 1:1 were added to the reaction vessel with the molar ratio of acid to alcohol of 1 to 1.2 to ensure alcohol end-groups. The reaction was performed in a nitrogen atmosphere and initiated by immersing the reaction vessel into a thermostated oil bath at 90 °C. The reaction temperature was subsequently increased to 190 °C at a rate of 5 °C/min. The catalyst, titanium isopropoxide (TIP), was thereafter added to start the deglycolization and the reaction temperature was increased to 220 °C. This second reaction step was allowed to proceed for 30 min under a reduced pressure at 0.35 mbar until the theoretical amount of water had been collected. After the polymerization was completed, the reaction mixture was cooled down to room temperature and the product was dissolved in 40 mL of chloroform and precipitated in ice-cold methanol. After decantation, the product was dried under vacuum to constant weight.

### 2.3. Grafting from Functionalization of the PVC Main Chain with Acrylic Acid or Crotonic Acid

Grafting of acrylic acid or crotonic acid to the PVC main chain to form PVC-AA and PVC-CA, respectively, was carried out in a two-necked reaction vessel equipped with a magnetic stirring bar using a modification of a previously reported method [31]. In addition, 5 g of PVC was first dissolved in cyclohexanone at 90 °C under rapid stirring until the polymer had dissolved completely. A suitable quantity of acrylic acid or crotonic acid and of the initiator benzoyl peroxide were subsequently added to the reaction vessel, and the reaction was performed for 3.5 h at 50 °C under vacuum (0.29 atm). A mass ratio of PVC to crotonic acidor acrylic acid of 1:0.1 (*w*/*w*) and a molar ratio of PVC:benzoyl peroxide of 1:1.4·× 10^−3^ were used. The reaction vessel was cooled to room temperature and the product was precipitated in ice-cold methanol. The grafted PVC was subsequently purified for 24 h by Soxhlet extraction using methanol at 90 °C, and the product was finally dried under vacuum to constant weight.

### 2.4. Film Preparation

Films of neat PVC, PVC-AA and PVC-CA and their 20% and 40% (*w*/*w*) blends with PBSA were prepared by solution casting. Each film was prepared by dissolving 1.25 g of the material in 50 mL of tetrahydrofuran at 60 °C with a high level of agitation. After the materials had dissolved completely, the solution was poured into a 9 cm diameter glass Petri dish. Each film was dried at room temperature in the fume hood for 3 days and then under vacuum for at least 3 days to constant weight before characterization. The films had a thickness of 120 ± 6 µm. The blend films were denoted as PVC/PBSA20, PVC/PBSA40, PVC-AA/PBSA20, PVC-AA/PBSA40, PVC-CA/PBSA20 and PVC-CA/PBSA40, where the number stands for % (*w*/*w*) of PBSA plasticizer.

### 2.5. Nuclear Magnetic Resonance (NMR)

^1^H NMR analyses of the polyesters as well as the grafted and coupled PVC materials were performed on a Bruker Avance DPX-400 Nuclear Magnetic Resonance spectrometer (Billerica, MA, USA) operating at 400 MHz. The analysis was performed at room temperature using chloroform-d (CDCl_3_) as a solvent for the analysis of polyester plasticizers and dimethyl sulfoxyde-d_6_ (DMSO) for grafted PVC.

### 2.6. Fourier Transform Infrared Spectroscopy (FTIR)

FTIR was utilized to characterize the synthesised plasticizers, to verify the grafting reactions, and to evaluate the miscibility of PVC and PBSA through the shift in the carbonyl group absorption band of the plasticizer in the blended products. FTIR spectra were recorded on a PerkinElmer Spectrum 2000 FTIR spectrometer (Norwalk, CT, USA) equipped with a single reflection attenuated total reflectance (ATR) accessory (golden gate) from Graseby Specac (Kent, UK). All FTIR spectra were obtained as a means of 5 samples and 16 individual scans at 4 cm^−1^ resolution.

### 2.7. Size Exclusion Chromatography (SEC)

The molecular weights of the synthesized polyester was measured by a Verotech PLGPC 50 Plus system with a PL-RI Detector and two Mixed-D (300 × 7.5 mm) columns from Varian (Palo Alto, CA, USA). The samples were injected with a PL-AS RT autosampler for PLGPC 50 Plus (Santa Clara, CA, USA), using chloroform as the mobile phase (1 mL/min, 30 °C). Polystyrene standards with a narrow molar mass distribution in the range from 580 to 400,000 g/mol were used for calibration. Corrections for the flow rate fluctuations were made using toluene as an internal standard. CirrusTM GPC Software (version 3.2) was used to process the data.

### 2.8. Differential Scanning Calorimetry (DSC)

DSC analysis of all the films was performed in a nitrogen atmosphere (50 mL/min) on a Metter Toledo DSC 820 (Stockholm, Sweden). Approximately 5–10 mg of the sample was enclosed into standard 40 µL aluminium cups. The PBSA plasticizer was analysed using the following temperature program: heating from 25 to 180 °C, holding isothermally at 180 °C for 2 min, then cooling from 180 to 80 °C and holding isothermally at −80 °C for 2 min, and finally heating again to 180 °C with 10 °C/min for all steps. The analysis of plain PVC, plasticized, and grafted PVC films were performed by heating from 25 to 200 °C, holding isothermally at 200 °C for 2 min, then cooling to −50 °C and holding isothermally for 2 min at −50 °C, and finally heating again until 200 °C under a nitrogen gas flow of 50 mL/min with a rate of 10 °C/min. The melting point was noted as the maximum value of the peaks from the second heating scan, and the glass transition temperature was taken as the midpoint of the glass transition from the second heating scan.

### 2.9. Tensile Testing

Tensile tests on the films were carried out using an Instron 5566 module (Instron, Stockholm, Sweden) according to the STM D638 10 standard. Strips with a width of 5 mm and a length of 50 mm were cut from the solution casted films, and 8–10 specimens were tested for each material. The measurements were performed with a 500 N load cell at a strain rate of 20 mm/min. Prior to testing, the samples were preconditioned at 23 °C and 50% Relative Humidity (RH) for 40 h according to the standard ASTM D618-08.

### 2.10. Thermo-Gravimetric Analysis (TGA)

TGA analyses of the PBSA plasticizers and of all films were performed on a Mettler-Toledo TGA/SDTA 851e (Mettler-Toledo, Stockholm, Sweden). Approximately 5–10 mg of each sample was put into a 70 μL ceramic cup without a lid. All of the samples were heated from 40 to 800 °C at the rate of at 10 °C/min under a 50 mL/min airflow in the furnace. The polyester plasticizers were also analyzed under 50 mL/min of N_2_.

## 3. Results and Discussion

**Scheme 1:**
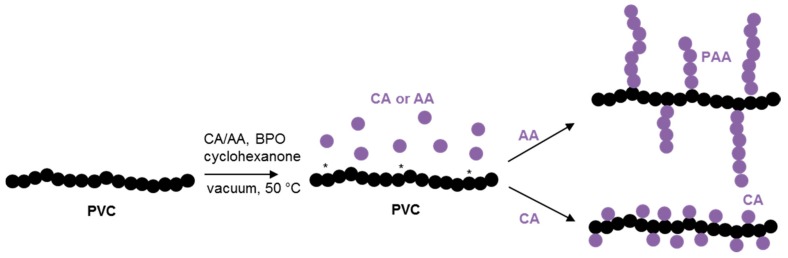
“Grafting-from” reaction of acrylic acid or crotonic acid from PVC in cyclohexanone at 50 °C using benzoyl peroxide as an initiator.

A straightforward grafting-from process to covalently attach AA or CA to the backbone of PVC was successfully used (Scheme 1 and Scheme S1). The achieved COOH functionalized PVC chains were then blended with an oligomeric biobased plasticizer (PBSA). The blend properties and the effect of functionalization were evaluated by comparing the material properties to those of plain PVC/PBSA blends and also to the properties of pure PVC, PVC-AA and PVC-CA films.

### 3.1. PVC Modification

Two different unsaturated acid monomers were used for the AA and CA grafting reactions to achieve the two envisioned distinctly different chemical structures for the grafted chains, Scheme 1. CA is unable or unlikely to homopolymerize due to high steric hindrance [32], while AA is well known to easily homopolymerize into high molecular weight grafts [33,34,35]. Since 10% (*w*/*w*) of monomer to PVC is used in both cases, the CA-grafted PVC should yield a more densely mono-functionalized PVC chain, whereas the AA-grafted PVC instead should yield PVC with fewer but longer AA grafts. To corroborate that CA does not homopolymerize, free radical polymerization using various reaction parameters such as AIBN or benzophenone as an initiator in various solvents (methanol, ethanol or water) and in bulk was performed and no polymer formation could be determined.

### 3.2. Functionalized and Plasticized Films

When designing the modification route, care was taken to ensure that the chemicals grafted or blended with PVC could be produced from renewable resources to as high a degree as possible, or as another option they could be produced by biopolymer recycling in order to create a non-toxic “greener” PVC. PBSA that functions as the actual oligomeric plasticizer can be synthesized from the renewable monomers 1,4-butanediol [36], succinic acid [36], and adipic acid [37], and, being an aliphatic polyester, it has an inherent degradability [38]. Although PBSA is not as commonly used as a PVC plasticizer as PBA [6,7,17,18], the incorporation of succinic acid into the copolymer should help to retain some of the stiffness inherent in the PVC. Due to the inherent chemical nature of PBSA, it will not crystallize in the PVC matrix, something commonly found for PBA, which should contribute to an overall increase in the softening effect of the plasticizer. In addition, CA is a degradation product formed during the thermal or microwave recycling of poly(3-hydroxybutyrate) [39,40,41], and AA is attainable from renewable resources [42]. The PBSA was synthesized using a step-growth polymerization with a slight excess of butanediol to ensure hydroxyl end-groups and low molecular weight. The number-average molecular weight of the synthesized PBSA was determined to be 2300 g/mol with a Đ = 2.9.

The properties of the functionalized PVC and PBSA (PVC-AA/PBSA, PVC-CA/PBSA) blends were compared to their analogous regular PVC/PBSA blends, to plain PVC and functionalized PVC (PVC-AA, PVC-CA) films to understand the influence of the functionalization. The composition of all the materials is given in Table 1.

### 3.3. FTIR and NMR Characterization of the Synthetic Products and Prepared Materials

**Figure 1:**
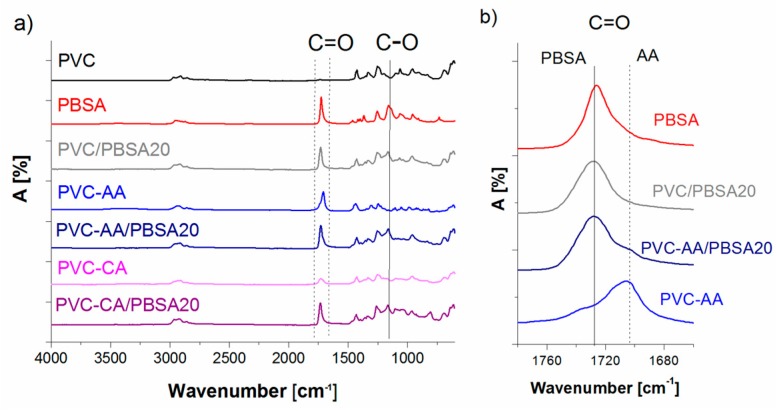
FTIR spectra of (**a**) neat PVC, neat PBSA plasticizer, PVC/PBSA20 blend, AA- and CA-grafted PVC alone and blended with 20% (*w*/*w*) PBSA plasticizer in the range of 4000 to 600 cm^−1^; (**b**) comparison of the carbonyl absorption band of the neat PBSA plasticizer, PVC/PBSA20, AA-grafted PVC blended with 0 and 20% (*w*/*w*) PBSA (PVC-AA and PVC-AA/PBSA) in the carbonyl group range (1800–1650 cm^−1^).

FTIR analysis of neat PVC showed the expected bands of C–Cl group at 613 cm^−1^, a C–H group at 1425 cm^−1^, and an alkyl C–H group at 2900 cm^−1^, Figure 1. The absence of the carbonyl group C=O absorption peak at 1700 cm^−1^ reveals that there is no plasticizer in the neat PVC and that the PVC is not oxidized. The neat PBSA plasticizer had a C=O absorption peak at 1731 cm^−1^, which can be compared to the PVC/PBSA20 and PVC/PBSA40 blends, which presented their C=O absorptions peak, originating from the PBSA, at 1728 and 1722 cm^−1^, respectively. This large shift in the carbonyl band has previously been demonstrated to be a strong indication of the miscibility between PVC and the plasticizers, while immiscible blends do not show a shift in the adsorption peak [43,44]. The miscibility between PVC and the aliphatic polyesters is achieved by interactions between the carbonyl group of the ester-linkage and the CHCl-group in PVC [43], and miscibility has been shown in the amorphous phase for all blend compositions. A relation between the miscibility and the CH_2_/COO ratio of the polyester has been proposed and a ratio of approximately 3–4 is suggested as the lower limit and 10–12 as the upper limit of miscibility [43,45,46]. The PBSA used here has a CH_2_/COO ratio of 3.5 and is hence in the lower range of the miscibility window.

In the AA-grafted PVC, a C=O band originating from the grafted AA was observed at 1706 cm^−1^, which is in agreement with previously reported values [47]. For CA-grafted PVC, a C=O band was observed at 1730 cm^−1^. The presence of the C=O absorption band originating from AA and CA, before and after Soxhlet purification with MeOH, supports the covalent attachment of the grafts [31]. For the AA-grafted films blended with PBSA (PVC-AA/PBSA20, PVC-AA/PBSA40, PVC-CA/PBSA20, and PVC-CA/PBSA40) broadened bands with shoulders due to the two different C=O groups were observed, corresponding to the bands observed for the neat PBSA plasticizer blends and for the AA- or CA-grafted PVC films, Figure 1b).

**Figure 2:**
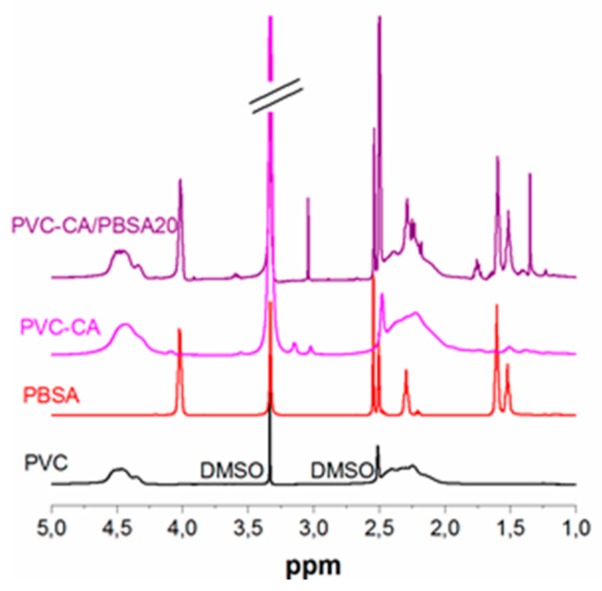
^1^H-NMR of PVC, PBSA plasticizer, PVC-CA and PVC-CA/PBSA20.

To further verify the functionalization of PVC, ^1^H-NMR of the CA-grafted PVC was compared to the spectra of plain PVC, (Figure 2). The disappearance of the vinyl groups from CA at 6.8, 5.8 and 1.8 ppm after the grafting reaction and purification in combination with the appearance of aliphatic protons (~1.8–0.5 ppm) originating from the CH3-groups indicate a successful reaction, although at lower relative concentrations compared to the feed concentration. The spectra of PBSA and PVC-CA/PBSA blend is also shown. A similar comparison for the AA-grafted materials could not be performed due to the insolubility of poly(acrylic acid) in DMSO.

### 3.4 Miscibility

The neat PVC films had a *T*_g_ value of 83.0 °C. The *T*_g_ of the grafted samples decreased by approximately 8 and 12 °C, i.e., to 75 and 71 °C for PVC-*g*-CA and PVC-*g*-AA, respectively. This indicates that CA or AA grafts extending from the main chain of PVC interrupted some of the intermolecular forces between the PVC chains. The larger reduction in the *T*_g_ of the PVC-*g*-AA compared with the PVC-*g*-CA further indicates that the main contributing factor for the larger change in *T*_g_ is that the grafted AA-chains are longer, yielding a larger chain mobility compared with that of the CA-functionality. This increased chain mobility would lead to a larger free volume and hence a larger reduction of the *T*_g_. The *T*_g_ region for PVC-*g*-AA is also broadened as compared to neat PVC and PVC-*g*-CA, which could indicate low or only partial miscibility between PVC and the long AA-grafts, as the *T*_g_ for low molar mass PAA is in the same range or slightly lower than the *T*_g_ of PVC. A second contributing factor to the reduction of the *T*_g_ is that more AA was grafted from the main chain of PVC.

**Figure 3:**
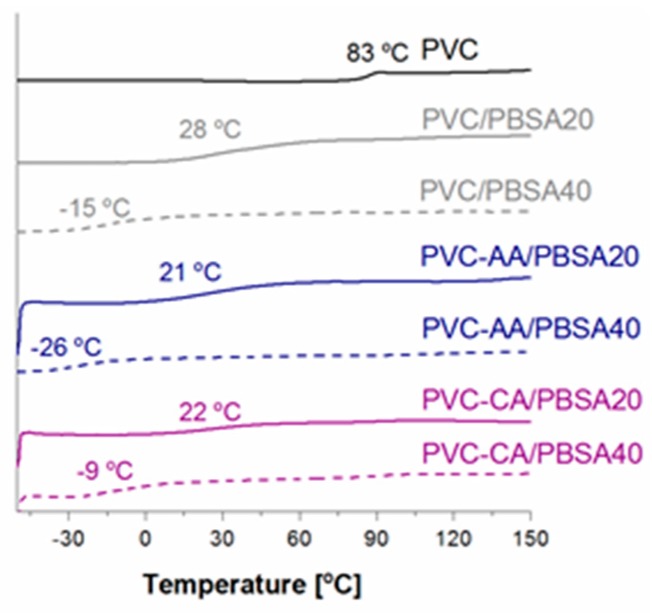
DSC thermograms showing the glass transitions of the different PVC films.

All the blended films portrayed one *T*_g_ that was reduced as compared to neat PVC and the plain grafted materials, a *T*_g_ that depended on the chemical composition. The *T*_g_ values measured by DSC were also in all cases lower than those calculated using the Fox equation, indicating complete miscibility between the plasticizer and the different PVC matrices. The largest reduction in *T*_g_ of around 100 °C was found for the grafted PVC films blended with 40% (*w*/*w*) PBSA plasticizer, Figure 3. The oligomeric PBSA plasticizers had a *T*_g_ = −49 °C and a *T*_m_ of 54.7 °C, which correspond well to the previously reported values [38]. No crystallization or melting of the PBSA plasticizer was observed in any of the films; hence, one homogeneous amorphous phase was seen for all materials.

A significant and linear reduction in *T*_g_ was observed from approximately 80 °C for the neat PVC to 28 and −15 °C for PVC plasticized with 20% and 40% (*w*/*w*) PBSA, respectively. This verifies the miscibility of the plasticizer in the PVC matrix and hence confirms the FTIR results. Comparison of the three films blended with 20% (*w*/*w*) PBSA plasticizer (PVC/PBSA20, PVC-AA/PBSA20, and PVC-CA/PBSA20) shows that films containing AA or CA-grafted PVC yield a lower *T*_g_ compared to the PVC/PBSA20 film, and therefore a similar overall reduction in temperature compared to the different starting glass transition temperature values. A similar phenomenon was observed for the corresponding films with 40% (*w*/*w*) PBSA plasticizer, except for PVC-CA/PBSA40. The lower overall reduction in *T*_g_ for the PVC-CA/PBSA40 to −9 °C does not follow the linear trend of reduction in *T*_g_ that all other samples portray and the *T*_g_ is unexpectedly high and more similar to that of the PVC/PBSA40 blend. This could therefore be an indication of the lower amount of monomeric CA units grafted to the main chain of PVC as compared to AA. This overall effect is most likely due to the synergistic effect of the PBSA plasticizer and increased PVC chain mobility caused by grafting.

Comparing the reduction in *T*_g_ for the CA- and AA-grafted PVC blends clearly shows a larger reduction for the AA-grafted films. This is again explained by the larger increase in the free volume due to the longer chains formed and larger amount of AA-grafted to the PVC backbone as compared to the monomeric CA units attached to the PVC backbone, and is in good agreement with what was observed for the plain PVC-AA and PVC-CA films.

### 3.5. Mechanical Properties

**Figure 4:**
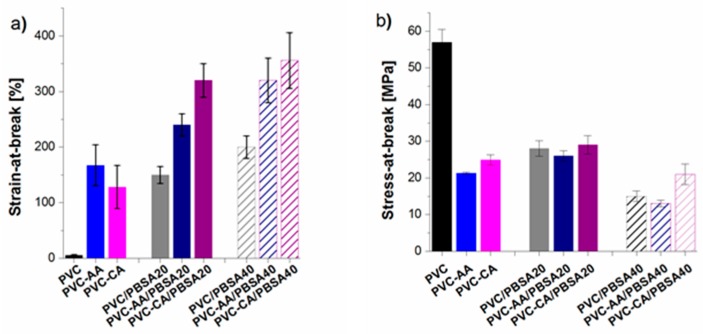
Mechanical properties of the PVC-based films as determine by tensile testing, (**a**) strain-at-break and (**b**) stress-at-break of the films.

The mechanical properties of the grafted PVC films were compared to their plain analogues to evaluate if the grafting functionalization increased the flexibility and improved the workability of the inherently brittle and rigid PVC, Figure 4. The strain-at-break of the PVC films was already significantly improved after the grafting from functionalization, increasing from 6% for plain PVC to 128% and 167% for PVC-CA and PVC-AA, respectively, i.e., an impressive and unexpected up to 28-fold increase in the strain-at-break was obtained after the grafting functionalization. In fact, the strain-at-break after grafting functionalization was at a similar level with the neat PVC films containing 20% (*w*/*w*) PBSA, and it is explained by the weakening of the secondary bonds between PVC chains after the functionalization.

All plasticized PVC-based films showed a significant increase in flexibility as compared to neat PVC, and at least a 25-fold increase in the strain-at-break was seen for all the materials, Figure 4. This high increase in the strain-at-break was coupled to a decrease in the stress-at-break, which decreased to 30%–50% of the original value recorded for plain PVC. This behaviour is typical for plasticized materials, especially when low molecular weight plasticizers are used.

The strain-at-break for the PBSA blends with neat and grafted PVC increased with an increasing amount of plasticizer. For the neat PVC blends, an increase was observed from 6% for the neat PVC to 150% and 200% for the blends with 20% and 40% (*w*/*w*) of PBSA, respectively. This increase in flexibility is in agreement with previous studies on PVC plasticized with PBA of varying architectures and molecular weights [6,7,17]. For the PBSA blends with AA- or CA-grafted PVC notably improved strain-at-break values were recorded at the same time as stress-at-break values were retained to a higher degree than in the traditional PVC/PBSA blend films with the same amount of plasticizer. This can be illustrated by the fact that the addition of 20% (*w/w*) of PBSA plasticizer to the grafted PVC-films resulted in clearly higher elongation as compared to the plain PVC/PBSA blend with 40% (*w*/*w*), i.e., twice the amount of plasticizer. As an example, the strain-at-break for PVC-CA/PBSA20 was 320%, as compared to 6%, 128%, 150% for PVC, PVC-CA and PVC/PBSA20, an over 50-fold increase as compared to plain PVC.

The reason behind this is most likely a combination of internal plasticization effect from the acid functionalization, breaking secondary bonding between PVC chains and the increased amount of interactions between the grafted chains and the plasticizer. For the grafted films, the secondary interactions consist not only of the interactions between the carbonyl group in the ester-linkage and the CHCl-group in PVC [39], but they also consist of the interactions between the carboxylic acids grafted to the PVC main chain and the PBSA plasticizer. The stronger secondary interactions between the PVC chains and plasticizer leads to improved miscibility through enthalpy reduction, which, in turn, could lead to more effective disruption of secondary interaction between PVC chains and increased elongation. For the PVC-based materials with 20% (*w*/*w*) of PBSA plasticizer, this is also coupled to a decrease in *T*_g_, which would contribute to a larger stress. The same was true when comparing the pure and AA-grafted materials containing 40% (*w*/*w*) plasticizers.

The different architectures of the grafts, i.e., the fewer but longer AA-grafts and the more numerous and short CA-grafts, had a significant effect on the mechanical properties of the materials. The highest elongation was found for the CA-grafted blend films (PVC-CA/PBSA). A probable explanation is found in the fewer and longer PAA chains attached to the PVC backbone that could result in a less homogeneous distribution of PBSA in the bulk. For the PVC-CA films, due to the inability of CA to homopolymerize, the shorter chains with more a homogeneous distribution of CA in the PVC bulk could lead to a more homogeneous distribution of also PBSA.

### 3.6 Thermal Stability

**Figure 5:**
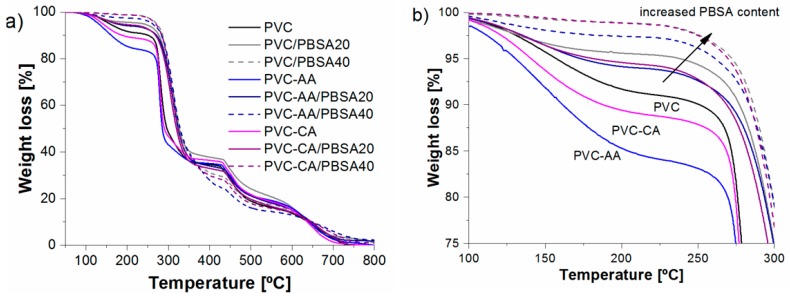
Thermal stability of neat PVC, PVC-AA, PVC-CA and the different PBSA blend films in the temperature range of (**a**) 50–800 °C and (**b**) 100–300 °C.

TGA analysis was performed to evaluate the effect of functionalization and blending on the thermal stability. The TGA results of the neat PVC and the grafted films show that the materials are mainly degraded by the two well-known stages of degradation characteristics for PVC. For neat PVC, the first main stage of degradation corresponded to the dehydrochlorination of PVC that commonly takes place in temperatures ranging from 220 to 370 °C [48]. The second stage corresponds to the degradation of the conjugated double bond structure formed during PVC dechlorination. The neat PVC film was stable until 160 °C, at which temperature a slow degradation was initiated; this degradation was followed by a higher degradation rate at temperatures ranging from 270 to 340 °C, corresponding with dehydrochlorination, after which the sample had lost 65% of its weight, as shown in Figure 5. The two grafted films, PVC-*g*-CA and PVC-*g*-AA, presented earlier onset of thermal degradation than did the neat PVC film. The lower thermal resistance induced by grafting either AA or CA to PVC was in line with results found for diethanolamine-functionalized PVC, where the grafted samples had a slightly reduced thermal stability compared with that of the virgin PVC [45]; however, the lower thermal resistance was in contrast with results of previous studies showing that grafting of PMMA and PS [49,50] onto the PVC main chain retarded the dehydrochlorination reaction. The main reason for this alteration is believed to be caused by the chemical structure of the grafted samples, where PMMA and PS are known to have a higher thermal resistance than PAA, which, in turn, has somewhat higher thermal resistance than PVC. Another contributing factor could be some detrimental effect on the thermal stability caused by the grafting protocol.

Additionally, the PVC-*g*-CA films exhibited a higher thermal resistance compared with that of PVC-*g*-AA. This is most likely caused by the longer grafts and abundance in AA-grafted PVC compared with those of PVC-*g*-CA and, hence, the larger abundance of functional groups in the grafted chains. Approximate amounts of grafted AA or CA in the films were calculated using TGA curves by taking the residual weight of the PVC-*g*-CA and PVC-*g*-AA at the onset temperature of dehydrochlorination and subtracting the weight of the neat PVC at the same temperature. It was estimated that approximately 2% (*w*/*w*) of CA and 6.8% (*w*/*w*) of AA was grafted to the main chain of PVC, supporting the hypothesis of the obtained chain structures, i.e., PVC-*g*-AA has fewer and longer PAA grafts and PVC-*g*-CA has a larger number of short (most likely monomeric) CA grafts.

Blending PBSA with plain or grafted PVC increased the initial thermal stability of the films with an increasing plasticizer content compared to neat PVC, Figure 5. During the initial stage, the films lost 60%–70% (*w*/*w*). The last stage corresponds to a simultaneous degradation of the PVC and the remaining plasticizer, Figure 5. The improvement in the thermal resistance during the dehydrochlorination stage is attributed to the fact that the polyester plasticizers such as PBSA can react with the hydrogen chloride (HCl) produced, forming carboxylic acids or chlorides acids [51,52] that have a less catalytic effect than HCl over the dehydrochlorination [51]. The larger the amount of plasticizer added, the more thermally stable the films were.

## 4. Conclusions

A combined grafting from functionalization and addition of PBSA bioplasticizers led to a superior plasticization effect in PVC/PBSA blends as determined by the increase in strain-at-break. Grafting CA or AA to the main chain of PVC generates carboxyl-functionalized and plasticized PVC of different chemical structures. The inability of CA to homopolymerize most likely resulted in PVC functionalized with monomeric units, while the higher reactivity and the inherent propensity of AA to homopolymerize would yield materials with grafted AA chains. Both types of grafted PVC portrayed a slight decrease in *T*_g_ and a substantial increase in elongation-at-break compared with those of neat PVC. The main influence of the chemical structure of the CA and AA grafts was observed in a slightly higher elongation-at-break for the materials with more abundantly incorporated AA grafts (6.8% (*w*/*w*)) compared with that of the materials with CA grafts (2.0% (*w*/*w*)), yet the results were on the same order of magnitude. Blending 20% (*w*/*w*) PBSA with PVC, PVC-AA or PVC-CA additionally increased the elongation at break 25-, 40- and 50-fold as compared to pristine PVC, respectively. To increase the sustainable character of PVC, the plasticizer and graft materials were chosen from monomers that can be produced from renewable resources or by feedstock recycling of biopolymer PHB. The desire to lower the carbon foot print of PVC and its additives while achieving increased plasticization efficiency were therefore combined.

## Figures and Tables

**Table 1:** Composition of the prepared PVC-based materials.

		Blend Composition		Grafting Feed	
Sample Name	Description	PVC/PVC-AA/PVC-CA [% *w*/*w*]	PBSA ^a^ [% *w*/*w*]	AA ^b^ [% *w*/*w*]	CA ^b^ [% *w*/*w*]
PVC	Neat PVC	100/0/0	0	-	-
PVC/PBSA20	Blend	80/0/0	20	-	-
PVC/PBSA40	Blend	60/0/0	40	-	-
PVC-AA	Grafted	-	-	10	-
PVC-AA/PBSA20	Grafted blend	0/80/0	20	10	-
PVC-AA/PBSA40	Grafted blend	0/60/0	40	10	-
PVC-CA	Grafted	-	-	-	10
PVC-CA/PBSA20	Grafted blend	0/0/80	20	-	10
PVC-CA/PBSA40	Grafted blend	0/0/60	40	-	10

^a^ Feed ration of PBSA with *M*_n_ = 2300 and Đ = 2.9 as determined using SEC with PS standards. ^b^ Feed ratio of AA and CA, respectively.

## Supplementary Materials

The following are available online at www.mdpi.com/2073-4360/9/3/84/s1, Scheme S1: Schematic reaction mechanism for the grafting of acrylic acid or crotonic acid to the main chain of PVC.
