# Supplementary material for: Superiorly Plasticized PVC/PBSA Blends through Crotonic and Acrylic Acid Functionalization of PVC
Source: Polymers (Basel). 2017 Mar 1;9(3):84. doi: 10.3390/polym9030084 (PMC6431921; doi:10.3390/polym9030084)
Supplement: Supplementary file 1 [file polymers-09-00084-s001.pdf]

Supplementary Material: Superiorly Plasticized PVC/PBSA Blends through Crotonic and Acrylic Acid Functionalization of PVC

Arturo Salazar Avalos, Minna Hakkarainen and Karin Odelius

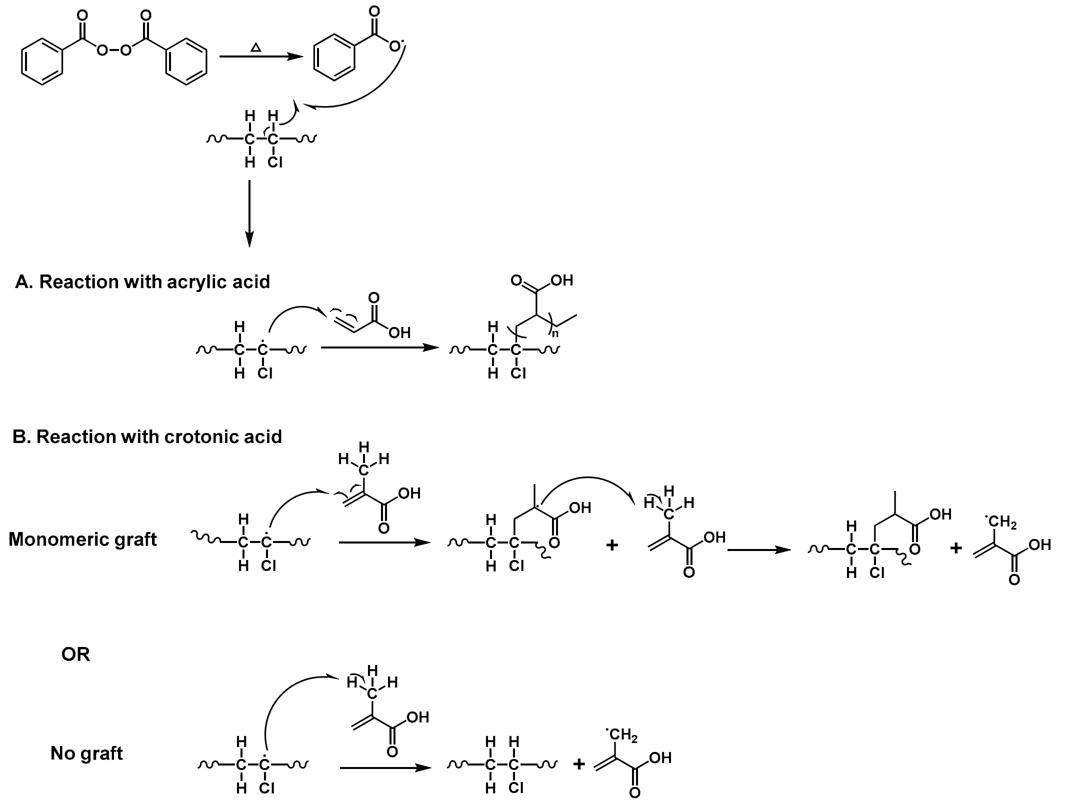

Scheme S1. Schematic reaction mechanism for the grafting of acrylic acid or crotonic acid to the main chain of PVC.
